# Current food trade helps mitigate future climate change impacts in lower-income nations

**DOI:** 10.1371/journal.pone.0314722

**Published:** 2025-01-03

**Authors:** Kushank Bajaj, Zia Mehrabi, Thomas Kastner, Jonas Jägermeyr, Christoph Müller, Florian Schwarzmüller, Thomas W. Hertel, Navin Ramankutty

**Affiliations:** 1 Institute for Resources, Environment and Sustainability, University of British Columbia, Vancouver, British Columbia, Canada; 2 School of Public Policy and Global Affairs, University of British Columbia, Vancouver, British Columbia, Canada; 3 Department of Environmental Studies, University of Colorado Boulder, Boulder, Colorado, United States of America; 4 Senckenberg Biodiversity and Climate Research Centre (SBiK-F), Frankfurt am Main, Germany; 5 NASA Goddard Institute for Space Studies, New York, NY, United States of America; 6 Center for Climate Systems Research, Columbia University, New York, NY, United States of America; 7 Member of the Leibniz Association, Potsdam Institute for Climate Impact Research (PIK), Potsdam, Germany; 8 Center for Global Trade Analysis, Purdue University, West Lafayette, IN, United States of America; MRC Unit The Gambia at LSHTM, GAMBIA

## Abstract

The risk of national food supply disruptions is linked to both domestic production and food imports. But assessments of climate change risks for food systems typically focus on the impacts on domestic production, ignoring climate impacts in supplying regions. Here, we use global crop modeling data in combination with current trade flows to evaluate potential climate change impacts on national food supply, comparing impacts on domestic production alone (domestic production impacts) to impacts considering how climate change impacts production in all source regions (consumption impact). Under 2°C additional global mean warming over present day, our analysis highlights that climate impacts on national supply are aggravated for 53% high income and 56% upper medium income countries and mitigated for 60% low- and 71% low-medium income countries under consumption-based impacts compared to domestic impacts alone. We find that many countries are reliant on a few mega-exporters who mediate these climate impacts. Managing the risk of climate change for national food security requires a global perspective, considering not only how national production is affected, but also how climate change affects trading partners.

## Introduction

Climate research in food systems has mainly focused on estimating impacts on the yields of major staple crops [1, 2]. However, a country’s food supply is not solely dependent on its own production. Food systems are highly interconnected and about one-fourth of calories produced are traded internationally [3]. Global agri-food trade enables low-income countries, often with lower per capita agricultural production, meet their nutritional needs [4], and therefore trade is deemed important for ameliorating the effects of climate change on food security [5]. However, trade also exposes food importing countries to cascading risks from trade embargoes and global price volatility during natural or man-made shocks [6, 7].

Prior studies have offered valuable insights into this topic. Baker et al. (2018) used a global agricultural economic model to explore the impact of global versus domestic climate change on the US agricultural sector, finding increased climate impacts under a global perspective [8]. Chen and Villoria (2019) identified higher price stability with increased import dependence across 27 net importing countries for maize [9]. Bren d’Amour and Anderson (2020) found that imports contribute to food supply instability in certain Global South countries [10]. Hedlund et al. (2022) examined the impact of climate change on food trade networks, observing high stability for wheat and rice networks but less for maize [11]. Jansenns et al. (2020) investigated various trade liberalization scenarios, highlighting significant benefits in addressing undernourishment through trade adaptation [5]. Other studies have also shock-tested food trade systems by examining the influence of removing crucial producing regions [12–16]. Despite these studies, much of the conversation on climate change and national impact assessments remains domestically focused [17, 18].

We introduce a complementary approach, tailored for national climate impacts assessments, to evaluate cross-border climate risks [17]. Our approach is simple and reproducible, and easily adapted by nations. We intentionally chose not to incorporate prospective trade flows that necessitate projecting global economy forward in time to keep our analysis simple and present a diagnosis based on current trade flows; other studies have used global trade economics models to explore the impacts of shifting trade patterns [5, 19]. The use of contemporary trade portfolios aligns with the recent shift in research focus (away from integrated assessment models) from projecting mid- or end-of-century future conditions to asking, "what would a 1/2/3°C warmer world do to current agricultural systems and food security?". This framing reduces the levels of uncertainty, allowing us to study the impacts of climate change without the added complexity of long-term economic projections [20]. We investigate how a nation’s total calorie supply is affected by climate impacts on its domestic production alone versus climate impacts over all its supplying regions (see conceptual framework in next section). These calories include those for human consumption, livestock feed, industrial purposes, and wasted. We do not consider nutrition, consumer acceptability, or household-level food security. Our analysis includes the three major staple crops–wheat, maize, and rice–that together contributed 43% of global calories produced and 87% of global cereal traded in 2010, and largely dominate trade related supply and price concerns under food crises. To align with policy discussions, we adopt a global mean warming approach, assessing climate impacts for future warming of 1°C and 2°C compared to present-day, rather than a time-based approach [21]. Our estimated crop yield impacts are influenced by changes in temperature, precipitation, radiation, and also associated carbon dioxide concentration. We focus on results for 2°C additional global mean warming in the main text (see Supplementary Information for results with 1°C additional future warming).

We use observed global bilateral trade flows of crop commodities corrected for re-exports [22, 23], modelled estimates of climate change impacts on crop yields [24], and spatial crop production data [25]. We also examine how key mega-exporters, those countries that three or more countries rely on for at least 10% of their calorie supply, influence climate impacts. Mega-exporters have a disproportionately large impact on global food supply, as demonstrated by the food price spikes related to the ongoing Ukraine-Russia conflict [26].

## Materials and methods

### Conceptual framework

Every country (y) consumes calories (C) from crop (i), which can be written as:

$$C_{y,i}=P_{y,i}-E_{y,i}+I_{y,i}$$ (1)

where *P*_*y*,*i*_ are the calories produced within the country, and E and I are the sum of all exports and imports of crop *y* to/from other countries.

We consider production net of exports (*P*_*y*,*i*_−*E*_*y*,*i*_) as production for domestic use P*_(y,i)_

$$C_{y,i}={P^{*}}_{y,i}+I_{y,i}$$ (2)

The impact of climate change on a nation’s consumption (ΔC) is:

$$\Delta C=\Delta P^{*}+\Delta I$$ (3)

Our analysis compares the three terms of Eq 3 in relative terms, i.e., we compare Δ*C*/*C* (*consumption impact*), ΔP*/P*(*domestic production impact*), and Δ*I*/*I* (*import impact*), (see S1 Text for the mathematical derivation relating these 3 terms and S2 Table for the summary of variables).

### Climate change impacts on crop productivity

We used estimates of climate impacts on global crop productivity from the latest process-based crop model simulations conducted as part of the Agricultural Model Intercomparison and Improvement Project’s (AgMIP) Global Gridded Crop Model Intercomparison (GGCMI) [24]. We used crop productivity data from 12 global crop models, forced by future climate simulations from 5 global climate models from the Coupled Model Intercomparison Project phase 6, using the Shared Socioeconomic Pathways (SSP) 585 climate scenario, bias-adjusted and downscaled by Inter-Sectoral Impact Model Intercomparison Project [27]. GGCMI provides 60 crop-climate model simulations per crop, running from pre-industrial times to 2100. For additional details see Jägermeyr et al 2021 [24].

### Global agri-food trade data

We used bilateral trade data for wheat, maize, and rice for the year 2010 from Schwarzmueller and Kastner 2022 [23], who designed an approach to link consumption impacts to origins of production. They used bilateral trade matrices and crop production data (from FAOSTAT) and traced crop supply in a country to where the crops were grown, correcting for re-exports of crop commodities. For instance, if maize is exported from France to the Netherlands where it is partly processed into maize germ oil, which is further exported to Austria, the corrected data will link calorie supply in Austria back to crop production in France. This data is widely used in estimating the environmental impacts of food consumption and telecoupling research [28–32]. We converted the bi-lateral trade data from tonnes to calories using conversation factors from GENuS [33].

### Domestic production impact

We estimated the domestic production impacts on calorie supply from climate change for each country from all 60 gridded *crop-climate model* simulations for the three crops. For each simulation we first estimated 10-year mean crop yields (at pixel level) around the year when global mean temperatures exceed 1°C, 2°C, and 3°C above pre-industrial levels in the respective climate model (see S1 Fig). We then assumed crop yields at 1°C global mean warming relative to pre-industrial as the reference as this roughly represents current global warming (exceedance years for 1°C warming range between 1999 and 2021 across our suite of climate models). Then we estimated gridded climate impacts as the percentage yield change with additional 1°C and 2°C future warming compared to our reference.

For each country *y*, crop *i*, crop-climate model *CCM*, and degree Celsius of additional global warming *w*, we estimated domestic production impact ${\Delta P^{*}}_{\left(y,i,CCM,w\right)}/{P^{*}}_{\left(y,i,CCM,w\right)}$ as production-weighted national average impacts denoted in percentage change terms (Eq 5; S1 Dataset):

$$\frac{{\Delta P^{*}}_{\left(y,i,CCM,w\right)}}{{P^{*}}_{\left(y,i,CCM,w\right)}}=\sum_{n=1}^{l}{CI}_{\left(i,w,CCM,n\right)}*\frac{A_{\left(i,n\right)}}{\sum A_{\left(i\right)}},$$ (5)

where *CI*_(*i*,*w*,*CCM*,*n*)_ is crop-model simulated climate impact (yield changes in percentage) for crop land pixel *n* and *A*_(*i*,*n*)_ is the total crop production in tonnes for pixel *n* from Monfreda et al. 2008 [25]. We assumed that harvested areas are not affected by climate change. We estimated domestic production impact for each country, crop and crop-climate model separately. Then we calculated country- and crop-specific ensemble median, and 20^th^ and 80^th^ percentile estimates (confidence intervals) across the 60 crop-climate model impact estimates. Note these are national level averages and so different impacts in different parts of the country could offset each other.

We estimated crop aggregated domestic production impact (${\bar{{\Delta P}^{*}}}_{\left(y,q,w\right)}/{\bar{P^{*}}}_{\left(y,q,w\right)}$) for each country *y*, *percentile q* and global warming *w*, as the weighted mean impact of warming on production for domestic use of the three crops (Eq 6; S1 Dataset):

$$\frac{{\bar{\Delta P^{*}}}_{\left(y,q,w\right)}}{{\bar{P^{*}}}_{\left(y,q,w\right)}}=\sum_{i=1}^{3}\frac{{\Delta P^{*}}_{\left(y,i,q,w\right)}}{{P^{*}}_{\left(y,i,q,w\right)}}*p_{\left(y,i\right)},$$ (6)

where *p*_(*y*,*i*)_ is production-share weights equal to the share of each crop to the total calories produced for domestic use from all three crops.

### Consumption and import impact

As with domestic production impact, we estimated the consumption and import impacts on supply for each country based on the three crops separately and for the crops aggregate. Consumption impact (${\Delta C}_{\left(y,i,q,w\right)}/C_{\left(y,i,q,w\right)}$) and import impact (${\Delta I}_{\left(y,i,q,w\right)}/I_{\left(y,i,q,w\right)}$) for a country are the weighted average production impacts across all supplying countries, including production for domestic-use for the former but excluding it for the latter (Eq 7 and Eq 8; S1 Dataset).

$$\frac{{\Delta C}_{\left(y,i,q,w\right)}}{C_{\left(y,i,q,w\right)}}=\sum_{x=1}^{m+1}\frac{{\Delta P^{*}}_{\left(x,i,q,w\right)}}{{P^{*}}_{\left(x,i,q,w\right)}}*\frac{z_{\left(x|y,i\right)}}{\sum z_{\left(y,i\right)}},$$ (7)

$$\frac{{\Delta I}_{\left(y,i,q,w\right)}}{I_{\left(y,i,q,w\right)}}=\sum_{x=1}^{m}\frac{{\Delta P^{*}}_{\left(x,i,q,w\right)}}{{P^{*}}_{\left(x,i,q,w\right)}}*\frac{z_{\left(x|y,i\right)}}{\sum z_{\left(y,i\right)}},$$ (8)

where *m* is the number of countries that consumer country *y* imports from, and *m+1* includes the supply from domestic production of the country in question. *z*_(*x*|*y*,*i*)_ are the calories contributed by a supplying region towards the total supply of the country in question.

Next, we computed crop-aggregated consumption (${\bar{\Delta C}}_{\left(y,q,w\right)}/{\bar{C}}_{\left(y,q,w\right)}$) and import impacts (${\bar{I}}_{\left(y,q,w\right)}/{\bar{I}}_{\left(y,q,w\right)}$) for each country using a similar weighted average approach as for crop-aggregated domestic impacts (S1 Dataset):

$$\frac{{\bar{\Delta C}}_{\left(y,q,w\right)}}{{\bar{C}}_{\left(y,q,w\right)}}=\sum_{i=1}^{3}\frac{{\Delta C}_{\left(y,i,q,w\right)}}{C_{\left(y,i,q,w\right)}}*N_{\left(y,i\right)},\;\mathrm{and}$$ (9)

$$\frac{{\bar{\Delta I}}_{\left(y,q,w\right)}}{{\bar{I}}_{\left(y,q,w\right)}}=\sum_{i=1}^{3}\frac{{\Delta I}_{\left(y,i,q,w\right)}}{I_{\left(y,i,q,w\right)}}*O_{\left(y,i\right)},$$ (10)

Where *N*_(*y*,*i*)_ and *o*_(*y*,*i*)_ are supply-share weights equal to the share of each crop to the total calories consumed and imported, respectively (see S2 Fig for a graphical explanation).

### Climate impact on national food supply

**Fig 1:**
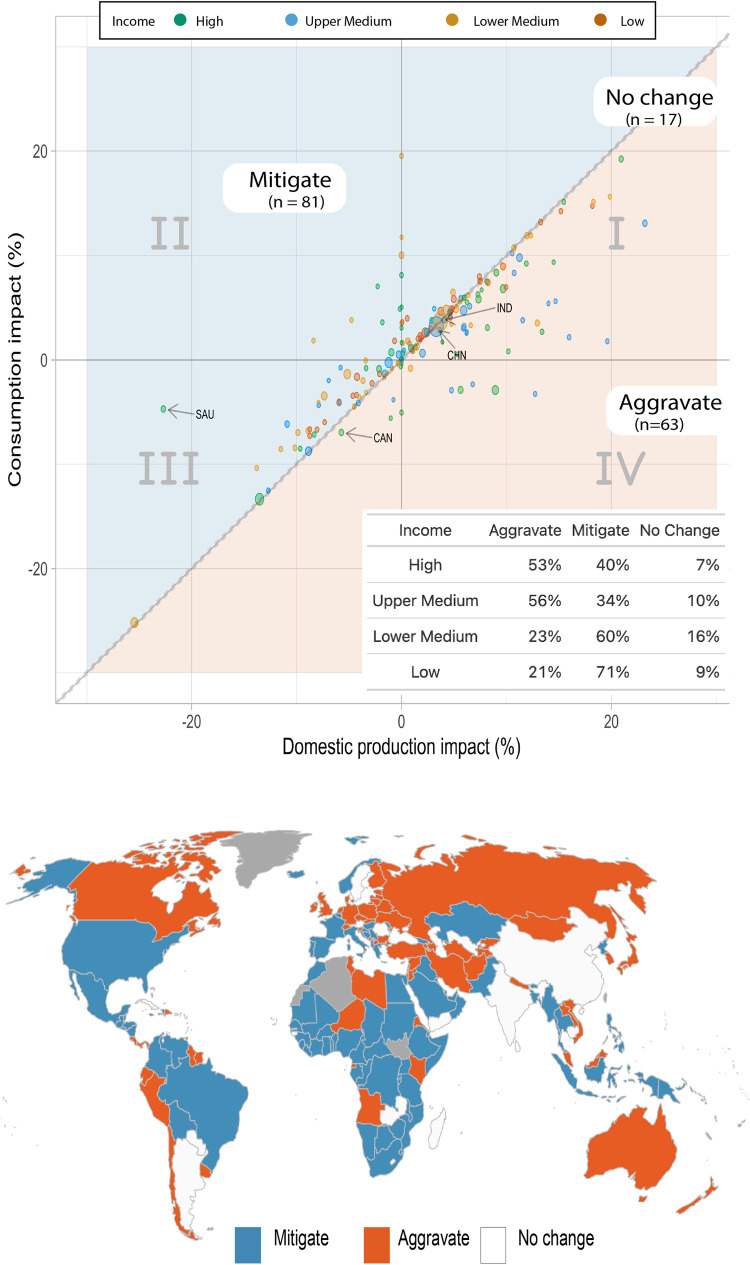
(Top panel). The climate impact on national food supply based on consumption and domestic perspective under additional 2°C global warming. We compare the crop-aggregated domestic climate impact (sensitivity of crop yields of wheat, maize, and rice to future warming) (x-axis, S4 Fig) to the consumption impact on calorie supply i.e., climate impacts on both domestic and overseas supplying regions (y-axis, S4 Fig). Positive impact values suggest more calories are projected to be available because of warming and negative impact values suggest fewer calories are projected to be available. Climate impact is aggravated when consumption impact increases the detrimental effect (lower loss to higher loss or gain to loss) or decrease the beneficial effect of climate change (from higher gain to lower gain) on the total calorie supply i.e., consumption impact is worse than domestic impact. Climate impact is mitigated if the opposite is the case, i.e., consumption impact decreases the detrimental impact (higher loss to lower loss) or increase the beneficial impact (lower gain to higher gain or loss to gain) on the total calorie supply. No effect on climate impact is considered when domestic impact and consumption impact are close to each other, where close is defined based on the lowest 5th percentile of the shortest distance between each point on the plot and the 1:1 line. The color of the data points corresponds to the World Bank income classification for the corresponding year and the size of the data points are scaled to 2010 national populations. Top panel (inset): We show the percentage of countries under each income class for which climate impacts are aggravated, mitigated, and shows no change under consumption impact. Bottom panel: The map shows the countries for which climate impact is mitigated (blue), aggravated (orange), and has no effect (white) on food supply for the three crops aggregated. For countries in gray, the impact estimates are not included. The map is generated using R version 4.2.1 and shapefiles sourced from the NaturalEarth project (naturalearthdata.com).

To evaluate how trade influences the climate impact on food supply for different countries (and populations), we compared their domestic production and consumption impact values. All countries with consumption impact higher than domestic production impact are classified as *aggravated* (i.e., climate impact on supply is heightened due to imports), and countries with consumption impact lower than domestic production impact as *attenuated* (i.e., climate impact on supply is lessened by imports). Countries are classified as having *no effect* when their domestic production and consumption impacts lie within the lowest 5th percentile of the perpendicular distance between each point and the 1:1 line of a scatter plot of the two variables (see Fig 1 top panel).

### Decomposition of cross-border effect

To better understand what drives the difference between consumption and domestic production impact, we estimated cross-border effect (Δ*C*/*C*−ΔP*/P*), which is composed of two components (Eq 11): 1) import dependence (*I*/*C*), and 2) difference between import impact and domestic production impact (see S1 Text for the full derivation of Eq 11; S1 Dataset).


$$\Delta C/C-\Delta P^{*}/P^{*}=I/C*(\Delta I/I-\Delta P^{*}/P^{*})$$ (11)

Thus, cross-border effect is higher for countries with large important dependence and for whom climate change impacts on imports exceed those on domestic production. It is important to distinguish between cross-border and import impacts. Cross-border impact highlights the per-calorie difference between a country’s consumption and its domestic production. In contrast, import impact reflects the climate burden of imports based on the quantities brought in from other countries. If a country’s consumption heavily relies on imports, the cross-border impact might closely resemble the import impact. In such cases, the distinction between the two may blur, leading to the perception that they are the same.

## Results

### Current food trade aggravates climate impacts on food supply for higher-income countries and mitigates it for lower-income countries

Under 2°C future global mean warming and 2010 trade portfolios, both domestic production and consumption impacts (aggregated for the three crops) are negative for 56 out of 162 countries (1.85 billion people; third quadrant in Fig 1, top panel). That is, climate change will reduce available calories for these countries irrespective of where they are sourced. These 56 countries constitute 49% of total global exported calories for the three crops and include large producers such as USA, Canada, and Brazil. Nevertheless, imports significantly mediate the impact of climate change for many countries–it aggravates the impact for 63 countries (1.3 billion people), but mitigates it for 81 countries (2.8 billion people). For example, Saudi Arabia experiences a –23% domestic production impact under 2°C warming, but this decreases to a consumption impact of –4.7%. In contrast, imports worsen climate impacts for Canada (–5.7% domestic production impact worsens to –6.9% consumption impact). Finally, for the most populous countries, China and India, consumption impacts are comparable to domestic impacts owing to their low import dependence for the three crops. We find that the effect of trade in mitigating climate impacts is muted under lower warming (S3 Fig). Under +1°C warming, the number of countries for which trade mitigates climate impacts reduces to 66 (2.3 billion people exposed) and aggravates increases to 78 (1.5 billion people exposed).

### Cross-border effects vary considerably by crop and countries

**Fig 2:**
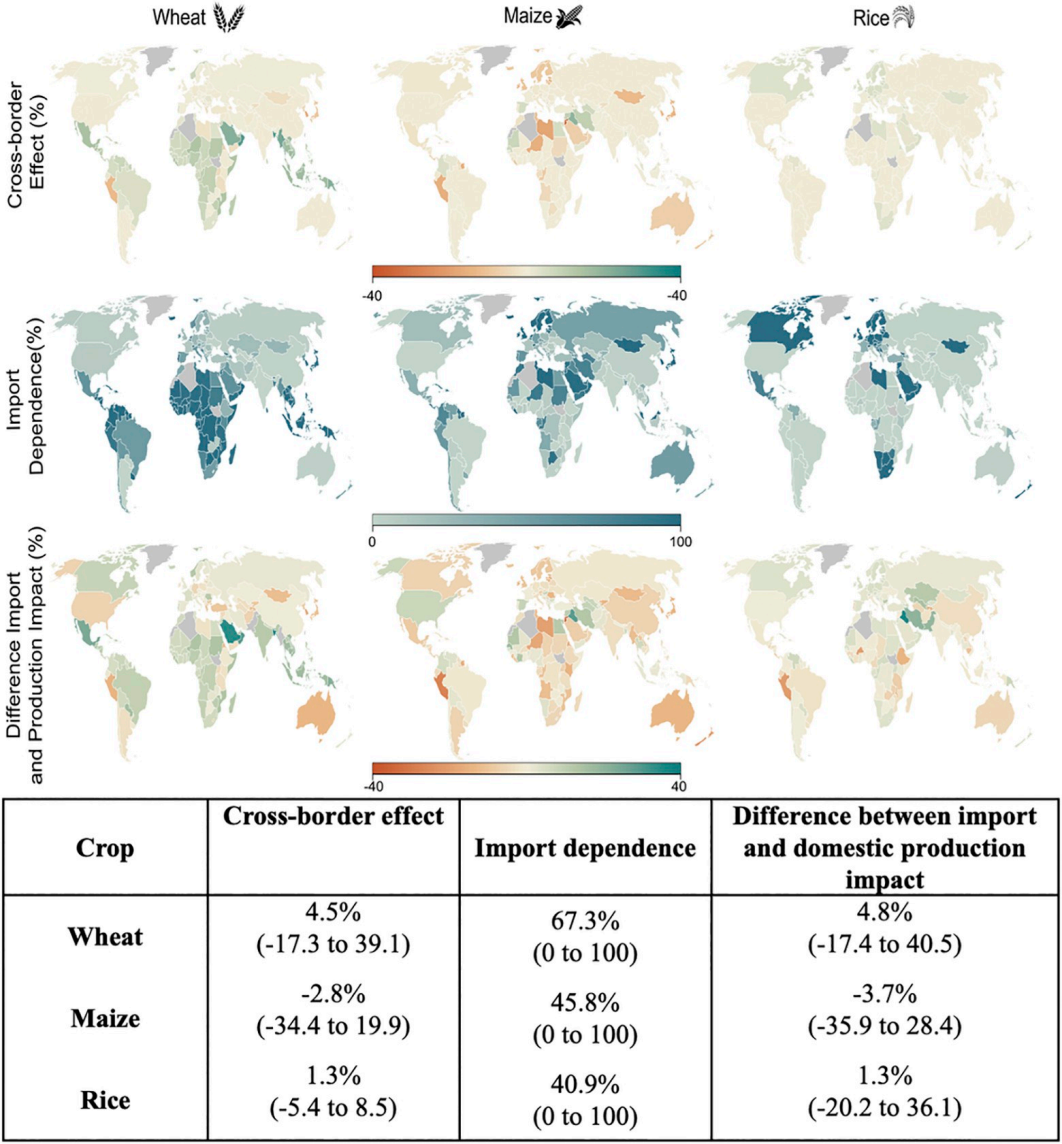
In this figure, we show the different factors influencing the consumption impact. For all the maps we consider 2°C mean global warming relative to the level of recent warming and fixed 2010 trade-portfolios. We divide this figure into three panels (a-c) horizontally, each panel containing three columns corresponding to the three crops- wheat, maize, and rice (from left to right). The panel a) shows the cross-border effect (as percentage impact per unit calorie) on total food supply of a particular country. The positive values mean that more calories will be available due to 2°C future mean global warming and negative values mean fewer calories (in percentage) will be available. The panel b) shows the import dependence (in percentage) of each country defined as the proportion of their crop-specific supply being sourced from other countries. In the panel c), we show the difference in import and domestic production impacts. For countries in gray, the estimates are not included. The inset table shows the global average (range) values for the variable shown in the three panels by crop. The maps are generated using R version 4.2.1 and shapefiles sourced from the NaturalEarth project (naturalearthdata.com).

Our decomposition results show that the factors determining cross-border effects vary considerably by crop and countries (Fig 2). The cross-border effect is interpreted as the impact on an average calorie of food consumed by a certain country compared with impact on a calorie of food produced for domestic-use by the same country. Positive values suggest impact on an average calorie consumed is less than impact on average tonne produced. Overall, beneficial cross-border effects are highest for wheat, both in magnitude and in terms of number of countries. Over 70% of countries have positive cross-border effects for wheat, with only a few (18 out of 161) having negative effects larger than 2%. The cross-border effects for maize are opposite to wheat. Most countries (except thirty-seven) have detrimental cross-border effects with over one third of large magnitude (greater than 2%). For rice, the cross-border effects are low in magnitude.

We decompose the cross-border effect into import dependence and the difference between import and production impacts (see methods section). We find the import dependence is highest for wheat, with most African and South Asian countries highly reliant on wheat imports. Both maize and rice have smaller import dependence compared to wheat (S2 Text). For import dependent countries, the difference between import and domestic production impact drives the cross-border effect (Eq 11; Fig 2).

With wheat, cross-border effects are largely positive for South Asian countries (Malaysia, Indonesia, Vietnam) due to a positive difference between import and domestic production impacts. Whereas, some east African countries (Uganda, Tanzania, Kenya) and east Asian countries (Japan, North and South Korea) have similar import dependence but find a negative difference between import and domestic production impacts. In the case of maize, for which the mean impact on importing partners is more severe and in most cases detrimental, this relationship is more pronounced and almost entirely true for most countries. For example, cross-border flows have a detrimental effect for countries with negative difference between import and domestic production impacts, like Libya, Niger, Jordon, Peru and Japan compared to countries with similar import dependence but with positive difference between import and domestic production impacts like Iraq, Egypt, Spain, Columbia, among others.

### Consumption-based perspective is especially important for import-dependent countries

**Fig 3:**
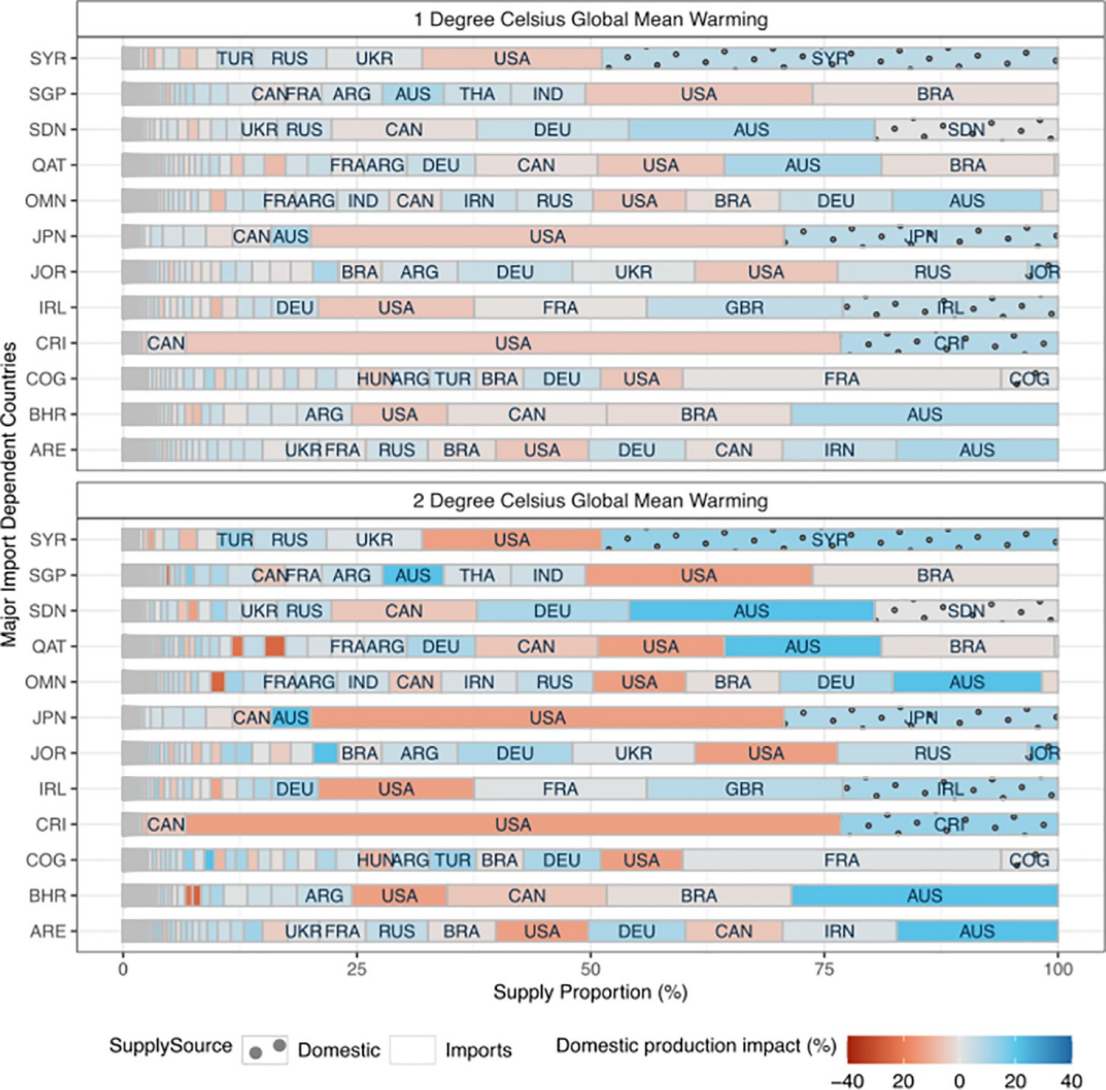
Visualizing how climate impacts are manifested for major import-dependent countries. The two panels correspond to 1°C and 2°C future mean global warming conditions relative to the level of recent warming (top & bottom). Each bar corresponds to a particular import-dependent country as labelled on the y-axis (see S1 Table for country codes). Each bar is made up of multiple stacks of different lengths and colors. Each stack corresponds to a particular country supplying calories to the importing country (labelled on the y-axis). The length of each stack (measured on the x-axis) within each bar corresponds to the proportion of total calories supplied by that supplying country to the importing country. The color of each stack represents the crop-aggregated (wheat, maize, and rice) climate change impact on domestic production of the supplying country under different warming scenarios.

We selected a few highly import-dependent countries to illustrate how climate impacts on national calorie supply is manifested (Fig 3). While some import-dependent countries (e.g., Japan, Costa Rica, Sudan, and Syria) rely on a few importing partners, others depend on a wider range of suppliers (such as Qatar, Oman, and the United Arab Emirates). For instance, both Japan and Costa Rica rely on the USA for more than 50% of their calorie supply from the three crops, a nation whose production is expected to be adversely affected by climate change. Further, climate impacts in the supplying countries can be positive or negative. For instance, Japan and Costa Rica experience negative import impacts from the USA, whereas Qatar, Sudan, Oman, Jordan, and the United Arab Emirates source a significant portion of their food supply from regions where climate change may increase production (such as France, Australia, Russia, Germany, and Ukraine) (Fig 3).

### Mega-exporters play a significant role in transmitting impacts

**Fig 4:**
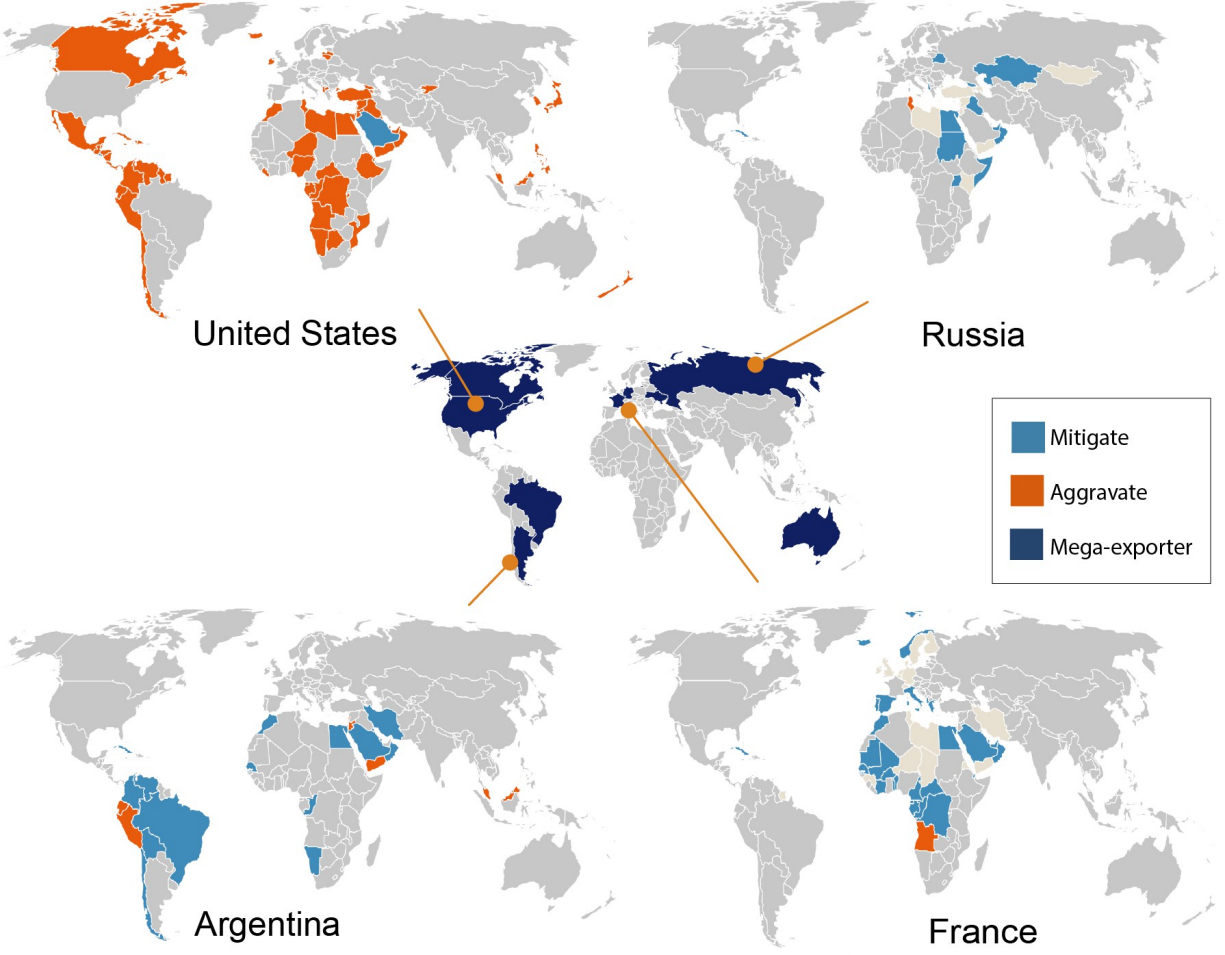
This figure shows for which countries mega-exporters aggravate (orange) or mitigate (blue) the climate impacts. The central map shows the various mega-exporters identified through our analysis. Each panel on the four corners shows a particular mega-exporter (panel label) and the role it plays in mediating the climate impacts for countries that source calories from it. For readability, we only show four of the nine mega-exporters and the importing partners that rely on a particular mega-exporter for at least 10% of their calorie supply from wheat, maize, and rice aggregated. The figure shows that 37 countries rely on the USA, 22 on France, 11 on Germany, and 11 on Russia for at least 10% of their total calorie supply. The maps are generated using R version 4.2.1 and shapefiles sourced from the NaturalEarth project (naturalearthdata.com).

We find that innumerable countries heavily rely on a handful of key mega-exporters, making them vulnerable to the climate impacts experienced by these mega-exporters. For the three crops aggregated, we identify nine mega-exporters: the USA, France, Germany, Russia, Australia, Brazil, Argentina, Ukraine, and Canada (Fig 4, S5 and S6 Figs). These mega-exporters contribute 77% of global calorie exports from the three crops. The USA in particular, but also Canada and Brazil are expected to face negative climate impacts. The other six mega-exporters are expected to benefit from combined yield improvements for the three staple crops under 2°C warming (see S6 Fig). These beneficial yield impacts are driven by wheat yield gains linked to increased carbon dioxide concentrations [24].

To comprehend the transmission of climate impacts, we compare the domestic impact of the mega-exporters with those of their importing partners (see Fig 4, S5 and S6 Figs). We find that the USA, Russia, Brazil, and Ukraine aggravate the supply impacts for more countries than they mitigate, while France, Germany, Brazil, Argentina, Australia, and Canada play a mitigating role for more countries than they aggravate. Additionally, the USA, which is expected to experience yield losses, has a strong aggravating impact on Japan, Central American countries, and a few Caribbean islands. These regions rely on the USA for over 50% of their calorie supply (indicated by the size of the bubbles in S6 Fig). In contrast, Argentina, Australia, and Canada mitigate impacts for most of the countries that depend on them (except for Malaysia and Peru in the case of Argentina, and Ecuador in the case of Canada). We also find certain countries are heavily dependent on multiple mega-exporters for a substantial portion of their calorie supply, which can either significantly aggravate or mitigate climate impacts (S7 Fig and S3 Text). Additionally, we examine the role of mega-exporters separately for wheat, maize, and rice, finding that their impact transmission varies across these three crops (S4 Text; S8–S10 Figs).

## Discussion

Transforming food systems to be resilient to supply disruptions from climate change requires resilience-building at multiple scales–at the farm scale by employing climate-resilient agriculture practices [34–36], but also at the national scale by managing reserves and trade portfolios [12]. In this paper, we examined how imports mediate the climate impacts on national food supply. We found that under 2°C future warming, imports largely mitigate impacts on food supply for low- and low medium income countries. For example, the climate impact on food supply is mitigated for Sudan when imports are incorporated into impact assessment. This is due to its high import dependence on Australia and Germany (mega-exporters), both of which have projected increases in wheat production under future climate change. On the contrary, imports aggravate climate impacts for Japan as a result of its high dependence on the USA, a mega-exporter expected to face negative climate impacts.

Our findings are consistent with an alternative definition of national consumption which does not exclude exports as it is considered to be within a nation’s control, for example, through an export embargo (S5 Text; S11 Fig). While our results consist of multiple sources of uncertainties, we evaluated one source of uncertainty based on the variation in impact estimates (20^th^ and 80^th^ percentile) across the sixty climate-crop model impact estimates. We find a larger variation in our climate impact estimates across these confidence intervals for a given degree of warming, compared to variation across the degrees of warming for a particular confidence interval (S3 Fig); these results reflect the findings of other studies that variation in yields simulated by different crop and climate models (which define our confidence intervals) for any given level of warming are greater than variation due to levels of warming [24, 37].

Our analysis examined climate impacts on calorie availability. But economic considerations may add further complexity to this picture. The economic status of countries, geographic distribution of climate impacts on crop yields, and the geographic structure of the global trade network determines winners and losers from increased global prices caused by natural or human-made production shocks [19]. High-income countries are generally better positioned to withstand price shocks from supply disruptions. More importantly, a rise in global prices benefits exporters of that commodity and hurt importers through changing “terms of trade” [19]. For example, supply disruptions from the ongoing Ukraine-Russia war, along with various compounding challenges, benefited other exporters (for example, wheat farmers of the USA) while import dependent (especially low and lower middle-income) countries battled high food prices to procure sufficient food [38]. Thus, it is critical to consider risks from both the availability and accessibility of food to design effective policies against food supply disruptions. This emphasizes the need for import-dependent countries to find a good balance between improving domestic food production capacity and diversifying their import portfolios to become more resilient to climate change. However, the capacities of countries to do so would vary substantially by income. Considering the ecological crises spurred by existing specialized and concentrated food production systems, diversification strategies may be beneficial for multiple objectives [34].

Our study contributes to the large body of literature on drivers of resilience in agri-food systems. While highly connected and diversified agri-food systems may be more resilient due to the pooling of risk [39–41], such systems can also be more vulnerable due to cascading or correlated crop failures in major food baskets [13, 42, 43]. Previous work [42] has emphasized two resilience principles—maintaining diversity and redundancy, and managing connectivity [44, 45]. Diversity in systems comprise not only variety but also balance in terms of the strength of different elements and disparity across these elements [45]. Our crop-aggregated consumption impact estimate incorporates diversity as a function of both calorie source diversity (across the three crops and their derived commodities) and import partner diversity (spatial diversity), and disparity as differences in climate impacts across crops and trading partners. Further, we measure connectivity by identifying the key actors, their exposure and sensitivity to climate change, and their interactions with other countries. We find that under the current food regime, mega-exporters play a large role in mediating climate risk. The network of such mega-exporters represents overly connected systems (which threaten resilience [45]) that have a large structure (many countries depend on them) and considerable strength (many countries relying on mega-exporters for a large share of food supply). We recommend that countries should take a systemic risk perspective [46] that considers the complex intertwined nature of the food systems.

There are several caveats to our analysis that should be noted. First, our analysis is limited to three crops because of the lack of robust estimates of crop-specific climate impacts and their economic ramifications for a larger variety of food items. Second, our analysis does not identify trade-offs between efficiency and resilience to supply disruptions as done by others [47] and this warrants future work. Third, our study does not allow for market or management related adaptation, either in the form of changing trade networks and demand due to rapid population growth or cropland allocation. Other economic [5, 19] and modelling [48–50] studies have shown the climate adaptation potential of these strategies. Our work could be expanded to examine the potential of shifting future trade networks on food supply risks, but incorporating future demand changes, especially in regions experiencing rapid population growth would be a necessary first step for effective future planning and policy design. Fourth, the climate impacts used in our analysis, despite being the latest efforts in the field, have large uncertainty [24]. Lastly, our global mean temperature framing of climate impact does not account for the differences in carbon dioxide concentration at these warming levels across the climate models. These variations can be large across climate models and strongly influence yield projections [51].

## Conclusion

The significance of cross-border climate impacts in food systems has grown with the increasing complexity and concentration of our global food systems. However, assessment of cross-border impacts has received limited attention in national climate assessments. Here we developed a simple analytical method to assess how climate impact on calorie supply of countries is attenuated or amplified by imports. We find that climate impacts are attenuated for a lot more low-income countries than high-income countries when food trade is incorporated in climate impact assessments. We show how mega-exporters play a disproportionately large role in mediating climate impacts. Our methodological approach is simple, adaptable, and easily reproducible to allow nations to evaluate cross-border climate impacts in food systems. Despite many limitations described above, our framework can assist policymakers in appraising current and future trade agreements and prioritizing strategies to enhance the resilience of food systems to future disruptions. Some such strategies include incentivizing sustainable and resilient farming practices, diversifying the domestic production of macro and micronutrients, managing food reserves and building redundancy, diversifying trade portfolios, and funding adaptation programs for trading partners.

## Supporting information

S1 Text Decomposition analysis. (DOCX)

S2 Text Global average cross-border effect on climate impact. (DOCX)

S3 Text Multiple mega-exporters and impact. (DOCX)

S4 Text Per-crop mega-exporters. (DOCX)

S5 Text Sensitivity analysis. (DOCX)

S1 Fig Global mean annual temperatures by climate models. (TIF)

S2 Fig Graphical representation of our per crop and crop aggregated consumption impact estimates. (TIF)

S3 Fig Sensitivity to crop and climate models. (TIF)

S4 Fig Production and consumption impact maps. (TIF)

S5 Fig Mega-exporters and climate impacts. (TIF)

S6 Fig Role of mega-exporters in mediating impacts. (TIF)

S7 Fig Network visualization. (TIF)

S8 Fig Role of mega-exporters in propagating impacts from supply of wheat. (TIF)

S9 Fig Role of mega-exporters in propagating impacts from supply of maize. (TIF)

S10 Fig Role of mega-exporters in propagating impacts from supply of rice. (TIF)

S11 Fig Market and central planner view. (TIF)

S1 Table List of ISO 3 country codes. (XLSX)

S2 Table Summary of variables used in the methods section of the main text. (XLSX)

S1 Dataset Contains the data used to conduct investigation and create figures. (XLSX)

## References

[1] Hertel TW. Viewpoint_ Climate impacts on agriculture_ Searching for keys under the streetlight. Food Policy. 2020;12.

[2] Davis KF, Downs S, Gephart JA. Towards food supply chain resilience to environmental shocks. Nature Food. 2021 Jan;2(1):54–65. doi: 10.1038/s43016-020-00196-3 37117650

[3] D’Odorico P, Carr JA, Laio F, Ridolfi L, Vandoni S. Feeding humanity through global food trade: D’ODORICO ET AL. Earth’s Future. 2014 Sep;2(9):458–69.

[4] Wood SA, Smith MR, Fanzo J, Remans R, DeFries RS. Trade and the equitability of global food nutrient distribution. Nature Sustainability. 2018 Jan;1(1):34–7.

[5] Janssens C, Havlík P, Krisztin T, Baker J, Frank S, Hasegawa T, et al. Global hunger and climate change adaptation through international trade. Nat Clim Chang. 2020 Sep;10(9):829–35. doi: 10.1038/s41558-020-0847-4 33564324 PMC7869491

[6] Headey D. Rethinking the global food crisis: The role of trade shocks. Food Policy. 2011 Apr 1;36(2):136–46.

[7] Martin W, Anderson K. Export Restrictions and Price Insulation During Commodity Price Booms. American Journal of Agricultural Economics. 2012;94(2):422–7.

[8] Baker JS, Havlík P, Beach R, Leclère D, Schmid E, Valin H, et al. Evaluating the effects of climate change on US agricultural systems: sensitivity to regional impact and trade expansion scenarios. Environ Res Lett. 2018 Jun;13(6):064019. doi: 10.1088/1748-9326/aac1c2 32153649 PMC7061454

[9] Chen B, Villoria NB. Climate shocks, food price stability and international trade: evidence from 76 maize markets in 27 net-importing countries. Environ Res Lett. 2019 Jan;14(1):014007.

[10] Bren d’Amour C, Anderson W. International trade and the stability of food supplies in the Global South. Environ Res Lett. 2020 Jun 23;15(7):074005.

[11] Hedlund J, Carlsen H, Croft S, West C, Bodin Ö, Stokeld E, et al. Impacts of climate change on global food trade networks. Environ Res Lett. 2022 Dec;17(12):124040.

[12] Reserves Marchand P. and trade jointly determine exposure to food supply shocks. Environ Res Lett. 2016;12.

[13] Puma MJ, Bose S, Chon SY, Cook BI. Assessing the evolving fragility of the global food system. Environ Res Lett. 2015 Feb 1;10(2):024007.

[14] Heslin A, Puma MJ, Marchand P, Carr JA, Dell’Angelo J, D’Odorico P, et al. Simulating the Cascading Effects of an Extreme Agricultural Production Shock: Global Implications of a Contemporary US Dust Bowl Event. Front Sustain Food Syst [Internet]. 2020 [cited 2021 Apr 7];4. Available from: https://www.frontiersin.org/articles/10.3389/fsufs.2020.00026/full.

[15] Gephart JA. Vulnerability to shocks in the global seafood trade network. Environ Res Lett. 2016;11.

[16] Distefano T, Laio F, Ridolfi L, Schiavo S. Shock transmission in the International Food Trade Network. Brunori G, editor. PLoS ONE. 2018 Aug 8;13(8):e0200639. doi: 10.1371/journal.pone.0200639 30089103 PMC6082532

[17] Carter TR, Benzie M, Campiglio E, Carlsen H, Fronzek S, Hildén M, et al. A conceptual framework for cross-border impacts of climate change. Global Environmental Change. 2021 Jul 1;69:102307.

[18] Challinor AJ, Adger WN, Benton TG. Climate risks across borders and scales. Nature Clim Change. 2017 Sep;7(9):621–3.

[19] Baldos ULC, Hertel TW, Moore FC. Understanding the Spatial Distribution of Welfare Impacts of Global Warming on Agriculture and its Drivers. American Journal of Agricultural Economics. 2019 Oct;101(5):1455–72.

[20] Christensen P, Gillingham K, Nordhaus W. Uncertainty in forecasts of long-run economic growth. Proceedings of the National Academy of Sciences. 2018 May 22;115(21):5409–14. doi: 10.1073/pnas.1713628115 29760089 PMC6003472

[21] Hausfather Z, Marvel K, Schmidt GA, Nielsen-Gammon JW, Zelinka M. Climate simulations: recognize the ‘hot model’ problem. Nature. 2022 May;605(7908):26–9. doi: 10.1038/d41586-022-01192-2 35508771

[22] Kastner T. Tracing distant environmental impacts of agricultural products from a consumer perspective. Ecological Economics. 2011;9.

[23] Schwarzmueller F, Kastner T. Agricultural trade and its impacts on cropland use and the global loss of species habitat. Sustain Sci [Internet]. 2022 May 12 [cited 2022 Jun 24]; Available from: 10.1007/s11625-022-01138-7.

[24] Jägermeyr J, Müller C, Ruane AC, Elliott J, Balkovic J, Castillo O, et al. Climate impacts on global agriculture emerge earlier in new generation of climate and crop models. Nat Food. 2021 Nov;2(11):873–85. doi: 10.1038/s43016-021-00400-y 37117503

[25] Monfreda C, Ramankutty N, Foley JA. Farming the planet: 2. Geographic distribution of crop areas, yields, physiological types, and net primary production in the year 2000: GLOBAL CROP AREAS AND YIELDS IN 2000. Global Biogeochem Cycles. 2008 Mar;22(1):n/a-n/a.

[26] Laber M, Klimek P, Bruckner M, Yang L, Thurner S. Shock propagation from the Russia–Ukraine conflict on international multilayer food production network determines global food availability. Nat Food. 2023 Jun;4(6):508–17. doi: 10.1038/s43016-023-00771-4 37322302

[27] Lange S. Trend-preserving bias adjustment and statistical downscaling with ISIMIP3BASD (v1.0). Geoscientific Model Development. 2019 Jul 17;12(7):3055–70.

[28] Kastner T, Chaudhary A, Gingrich S, Marques A, Persson UM, Bidoglio G, et al. Global agricultural trade and land system sustainability: Implications for ecosystem carbon storage, biodiversity, and human nutrition. One Earth. 2021 Oct 22;4(10):1425–43.

[29] Dalin C, Wada Y, Kastner T, Puma MJ. Groundwater depletion embedded in international food trade. Nature. 2017 Mar;543(7647):700–4. doi: 10.1038/nature21403 28358074 PMC7427584

[30] Agricultural Pendrill F. and forestry trade drives large share of tropical deforestation emissions. Global Environmental Change. 2019;10.

[31] Li Y, Zhong H, Shan Y, Hang Y, Wang D, Zhou Y, et al. Changes in global food consumption increase GHG emissions despite efficiency gains along global supply chains. Nat Food. 2023 Jun 15;1–13. 37322300 10.1038/s43016-023-00768-z

[32] Hoang NT, Taherzadeh O, Ohashi H, Yonekura Y, Nishijima S, Yamabe M, et al. Mapping potential conflicts between global agriculture and terrestrial conservation. Proceedings of the National Academy of Sciences. 2023 Jun 6;120(23):e2208376120. doi: 10.1073/pnas.2208376120 37252987 PMC10266011

[33] Smith MR, Micha R, Golden CD, Mozaffarian D, Myers SS. Global Expanded Nutrient Supply (GENuS) Model: A New Method for Estimating the Global Dietary Supply of Nutrients. PLOS ONE. 2016 Jan 25;11(1):e0146976. doi: 10.1371/journal.pone.0146976 26807571 PMC4726504

[34] Hertel T, Elouafi I, Tanticharoen M, Ewert F. Diversification for enhanced food systems resilience. Nat Food. 2021 Nov;2(11):832–4. doi: 10.1038/s43016-021-00403-9 37117513

[35] Lipper L. Climate smart agriculture: building resilience to climate change. 1st edition. New York, NY: Springer Science+Business Media; 2017.

[36] Campbell BM, Hansen J, Rioux J, Stirling CM, Twomlow S, (Lini) Wollenberg E. Urgent action to combat climate change and its impacts (SDG 13): transforming agriculture and food systems. Current Opinion in Environmental Sustainability. 2018 Oct 1;34:13–20.

[37] Müller C, Franke J, Jägermeyr J, Ruane AC, Elliott J, Moyer E, et al. Exploring uncertainties in global crop yield projections in a large ensemble of crop models and CMIP5 and CMIP6 climate scenarios. Environ Res Lett. 2021 Feb;16(3):034040.

[38] FAO. Impact of the Ukraine-Russia conflict on global food security and related matters under the mandate of the Food and Agriculture Organization of the United Nations (FAO) [Internet]. 2022 Apr [cited 2022 Apr 27]. (Hundred and sixty-ninth Session). Report No.: CL 169/3. Available from: https://www.fao.org/3/ni734en/ni734en.pdf.

[39] Feng X, Hayes D. Diversifying systemic risk in agriculture. Agricultural Finance Review. 2016 Jan 1;76(4):512–31.

[40] Sartori M, Schiavo S. Connected we stand: A network perspective on trade and global food security. Food Policy. 2015 Nov 1;57:114–27.

[41] Renard D, Tilman D. National food production stabilized by crop diversity. Nature. 2019 Jul;571(7764):257–60. doi: 10.1038/s41586-019-1316-y 31217589

[42] Kummu M, Kinnunen P, Lehikoinen E, Porkka M, Queiroz C, Röös E, et al. Interplay of trade and food system resilience: Gains on supply diversity over time at the cost of trade independency. Global Food Security. 2020 Mar;24:100360.

[43] Tu C, Suweis S, D’Odorico P. Impact of globalization on the resilience and sustainability of natural resources. Nat Sustain. 2019 Apr;2(4):283–9.

[44] Biggs R, Schlüter M, Schoon ML, editors. Principles for building resilience: sustaining ecosystem services in social-ecological systems. Cambridge: Cambridge University Press; 2015. 290 p.

[45] Biggs R, Schlüter M, Biggs D, Bohensky EL, BurnSilver S, Cundill G, et al. Toward Principles for Enhancing the Resilience of Ecosystem Services. Annual Review of Environment and Resources. 2012;37(1):421–48.

[46] Bernard de Raymond A, Alpha A, Ben-Ari T, Daviron B, Nesme T, Tétart G. Systemic risk and food security. Emerging trends and future avenues for research. Global Food Security. 2021 Jun 1;29:100547.

[47] Karakoc DB, Konar M. A complex network framework for the efficiency and resilience trade-off in global food trade. Environ Res Lett. 2021 Sep;16(10):105003.

[48] Franke JA, Müller C, Minoli S, Elliott J, Folberth C, Gardner C, et al. Agricultural breadbaskets shift poleward given adaptive farmer behavior under climate change. Global Change Biology. 2022;28(1):167–81. doi: 10.1111/gcb.15868 34478595

[49] Sloat LL, Davis SJ, Gerber JS, Moore FC, Ray DK, West PC, et al. Climate adaptation by crop migration. Nat Commun. 2020 Mar 6;11(1):1243. doi: 10.1038/s41467-020-15076-4 32144261 PMC7060181

[50] Cui X, Zhong Z. Climate change, cropland adjustments, and food security: Evidence from China. Journal of Development Economics. 2024 Mar 1;167:103245.

[51] Schleussner CF, Deryng D, Müller C, Elliott J, Saeed F, Folberth C, et al. Crop productivity changes in 1.5°C and 2°C worlds under climate sensitivity uncertainty. Environ Res Lett. 2018 May;13(6):064007.

