# Supplementary material for: Current food trade helps mitigate future climate change impacts in lower-income nations
Source: PLoS One. 2025 Jan 3;20(1):e0314722. doi: 10.1371/journal.pone.0314722 (PMC11698460; doi:10.1371/journal.pone.0314722)
Supplement: S1 Text — (DOCX) [file pone.0314722.s001.docx]

1. **Decomposition analysis**

To better understand what drives the difference between consumption and domestic production impact, we estimated a cross-border effect (∆C/C – ∆$P^{*}$/$P^{*}$), which is composed of two components (eq. 11 of the Main Text): 1) import dependence *(I/C),* and 2) difference between import impact and domestic production impact. We provide the derivation of our decomposition method below:

Suppose a country A, imports from r number of countries in addition to its production for domestic use ($P^{*}$).

Governing equation:

$\Delta C= \Delta P^{*}+ \Delta I$ (1)

Where *C*: consumption, $P^{*}$: production for domestic use, $I$: total overseas imports, and $\Delta$: change with climate change.

Dividing eq. 1 by C, we get:

$$\frac{\Delta C}{C} = \frac{\Delta P^{*}}{C}+ \frac{\Delta I}{C}$$

Therefore:

$$\frac{\Delta C}{C}-\frac{\Delta P^{*}}{P^{*}} = \frac{\Delta P^{*}}{C}+\frac{\Delta I}{C}-\frac{\Delta P^{*}}{P^{*}}$$

$$= \Delta P^{*}\left( \frac{1}{C}- \frac{1}{P^{*}} \right)+ \frac{\Delta I}{C}* \frac{I}{I}$$

$$= \Delta P^{*}\left( \frac{P^{*}-C}{P^{*}C} \right)+ \frac{\Delta I}{I}* \frac{I}{C}$$

$$= \frac{\Delta P^{*}}{P^{*}}\left( \frac{-I}{C} \right)+ \frac{\Delta I}{I}* \frac{I}{C}$$

$$= \frac{I}{C} \left( \frac{\Delta I}{I}- \frac{\Delta P^{*}}{P^{*}} \right)$$

Where, $\frac{\Delta C}{C}-\frac{\Delta P^{*}}{P^{*}}$ is the cross-border effect, $\frac{I}{C}$is the import dependence, and $\left( \frac{\Delta I}{I}- \frac{\Delta P^{*}}{P^{*}} \right)$ is the difference between import impact and production impact.
