# Supplementary material for: Current food trade helps mitigate future climate change impacts in lower-income nations
Source: PLoS One. 2025 Jan 3;20(1):e0314722. doi: 10.1371/journal.pone.0314722 (PMC11698460; doi:10.1371/journal.pone.0314722)
Supplement: S2 Text — (DOCX) [file pone.0314722.s002.docx]

1. **Global average cross-border effect on climate impact**

To estimate the global average cross-border effect on climate impact per crop, we average each factor described in the decomposition analysis (in the main section of the paper) across all countries. Although accurate, some global average estimates (described in the Figure 2 inset in the main text) can be misleading and require further explanation. First, the global average import dependence values should not be confused with proportion of global production that is traded. For example, globally only 5% to 6% of rice production is traded internationally. However, our estimate of global-average import dependence for rice is 40.9% (Fig. 2 inset in the main text). This high import dependence estimate is a result of numerous small countries importing high proportion of their food. This is particularly true in case of rice and wheat which is produced in a handful countries and most other countries dependent on cross-border flows for their supply.
