# Supplementary material for: Current food trade helps mitigate future climate change impacts in lower-income nations
Source: PLoS One. 2025 Jan 3;20(1):e0314722. doi: 10.1371/journal.pone.0314722 (PMC11698460; doi:10.1371/journal.pone.0314722)
Supplement: S3 Text — (DOCX) [file pone.0314722.s003.docx]

1. **Multiple mega-exporters and impact**

Certain countries are heavily dependent on multiple mega-exporters for a substantial portion of their calorie supply, which can either significantly aggravate or mitigate climate impacts (Main Text Fig. 5). By examining calorie flows constituting more than 15% of the national calorie supply, we observe mixed results (Main Text Fig. 3). For instance, both the United States and France aggravate the impact on Ireland, Yemen, Niger, and Israel. Similarly, Brazil and Argentina, and Argentina and the United States collectively aggravate impacts on Malaysia, Peru, and Costa Rica, respectively. In contrast, the United States and France jointly mitigate impacts for Gabon; Australia and Brazil do so for Oman, Qatar, and Kuwait; Argentina and Brazil for Kuwait; Brazil and the United States for Saudi Arabia; France and Brazil for Angola; the United States and Canada for Venezuela, and Australia and Canada for Jamaica, United Arab Emirates and, Sudan. We also corroborate our previous findings that a larger and stronger network is associated with mega-exporters like the United States and France, with the former aggravating impacts for the largest number of countries.
