# Supplementary material for: Current food trade helps mitigate future climate change impacts in lower-income nations
Source: PLoS One. 2025 Jan 3;20(1):e0314722. doi: 10.1371/journal.pone.0314722 (PMC11698460; doi:10.1371/journal.pone.0314722)
Supplement: S4 Text — (DOCX) [file pone.0314722.s004.docx]

1. **Per-crop mega-exporters**

Our analysis suggests that in cases of maize all mega-exporters, except Argentina, will produce fewer calories in the; while for wheat and rice most mega-exporters will experience higher productivity. In the case of wheat, we show all mega-exporters, except Ukraine and Russia, mitigate impact for many countries that rely on them for a large share of their calorie supply; while Russia and Ukraine aggravate impacts for more countries (demonstrated by the number of lollipops in each panel, the size, and the color of the bubble in the S6 Fig.). In case of maize, we find all mega-exporters, except Argentina, aggravate the impacts for most countries, with distortional large number of countries associated with USA, France and Brazil (S7 Fig.). For rice, the impact is relatively low compared to wheat and maize for the mega-exporters (as demonstrated by the x-axis in S8 Fig.). Most mega-exporters will experience higher rice productivity under 2°C additional warming, with China with the highest productivity gains. Except United States, most producing regions mitigate the impacts. However, it is important to note that domestic production impacts for sourcing regions that do not grow rice are considered zero here and comparing these with the domestic impacts of mega-exporters are not realistic.
