# Supplementary material for: Current food trade helps mitigate future climate change impacts in lower-income nations
Source: PLoS One. 2025 Jan 3;20(1):e0314722. doi: 10.1371/journal.pone.0314722 (PMC11698460; doi:10.1371/journal.pone.0314722)
Supplement: S5 Text — (DOCX) [file pone.0314722.s005.docx]

1. **Sensitivity analysis**

We carry out a sensitivity analysis to discuss the robustness of our findings. We check how our cross-border effect estimates vary with a different definition of national food consumption. In the main text, we estimate consumption impact estimates and decompose them based on the decentralized market perspective in which the basic pattern of exports and imports is governed by pre-existing business relationships. In the market view, we define consumption as the sum of production and imports minus exports – assuming that trade flows will persist in the context of climate change. We compare the cross-border effect estimates based on this definition with an alternate estimate based on central-planner view on national food consumption. The central planner view assume consumption as the sum of total production and overseas imports; exports are not excluded and are assumed to be part of the total production. From a risk perspective, the central planner view assumes a country can control what it does with its exports i.e., in a moment of crisis it can impose an export embargo.

To compare the two different perspectives, we plot the cross-border effect estimated from the decomposition analysis for both market and central planner view with respect to exports (S9 Fig.). We find that the cross-border effect is not sensitive to the central planner view.
