# Supplementary figures and images for: Current food trade helps mitigate future climate change impacts in lower-income nations
Source: PLoS One. 2025 Jan 3;20(1):e0314722. doi: 10.1371/journal.pone.0314722 (PMC11698460; doi:10.1371/journal.pone.0314722)

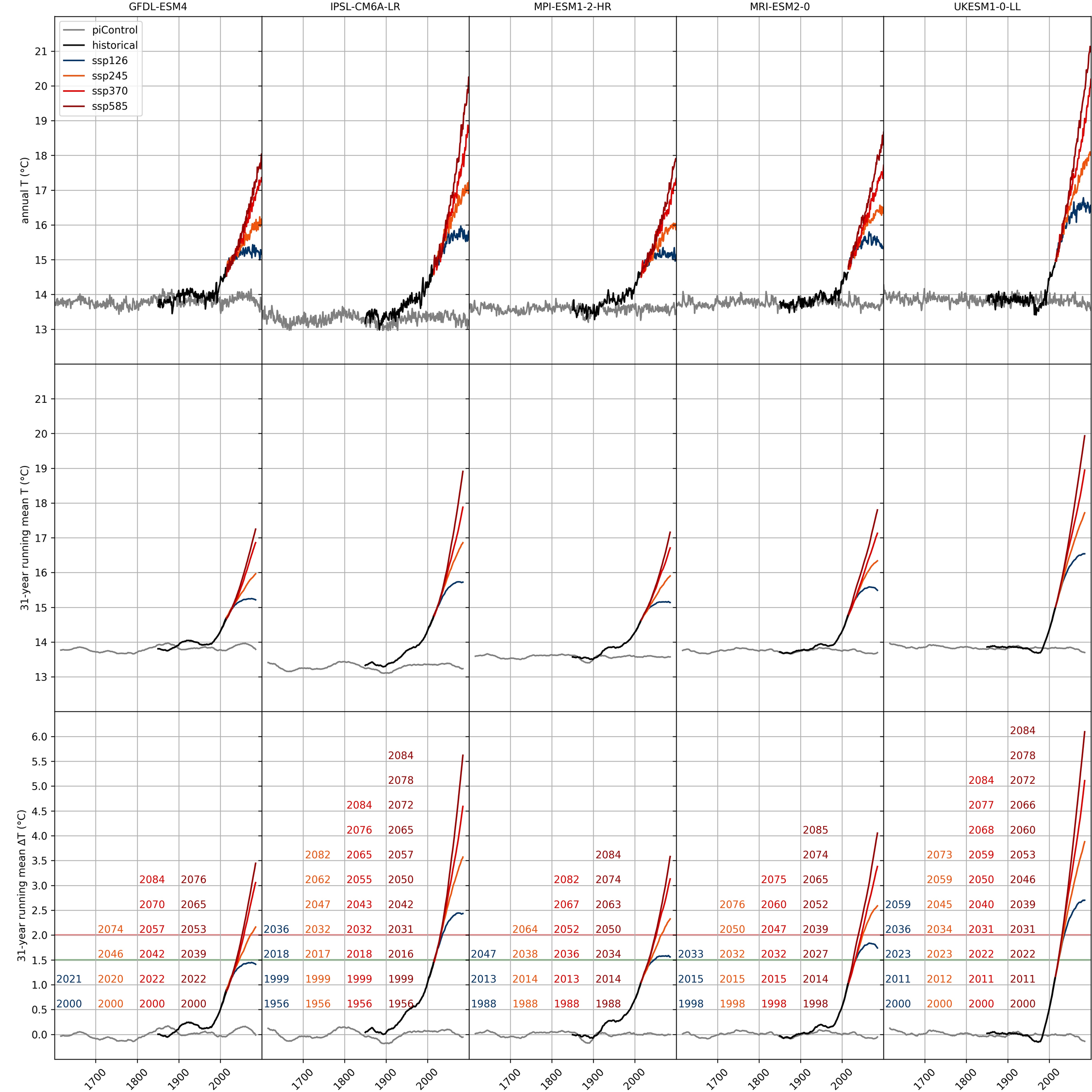

Supplement: S1 Fig — (TIF) [file pone.0314722.s006.tif]

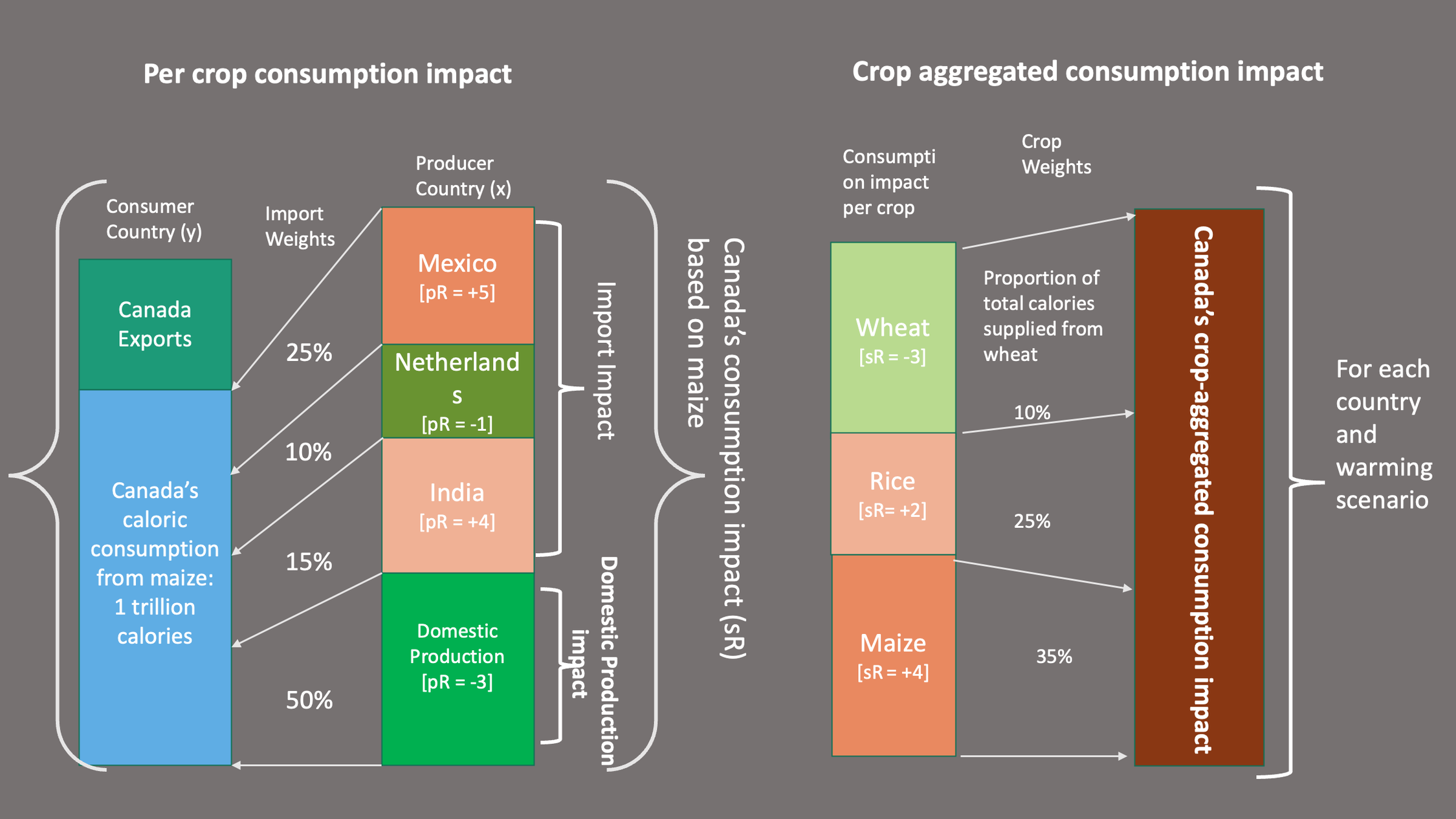

Supplement: S2 Fig — (TIF) [file pone.0314722.s007.tif]

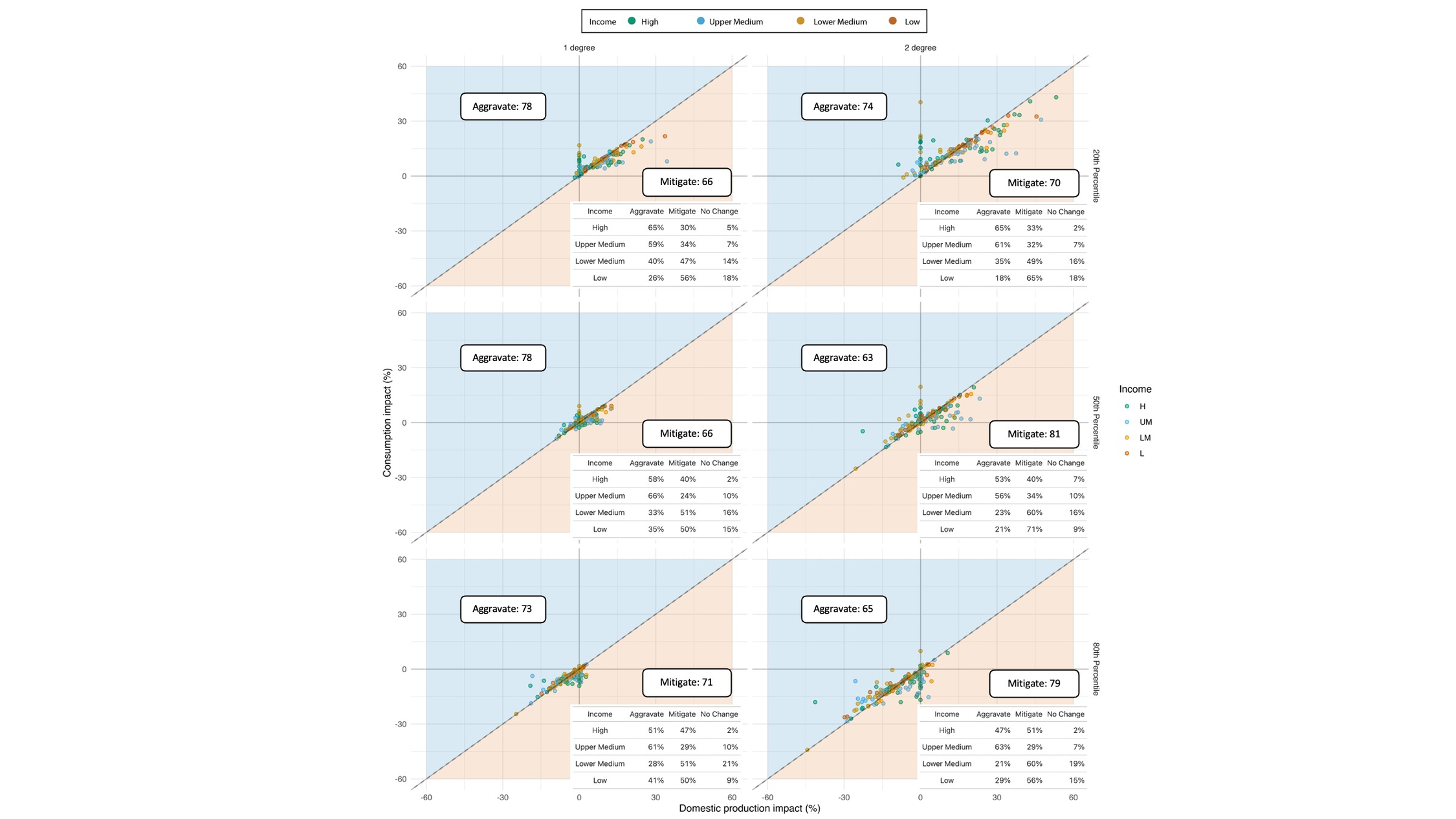

Supplement: S3 Fig — (TIF) [file pone.0314722.s008.tif]

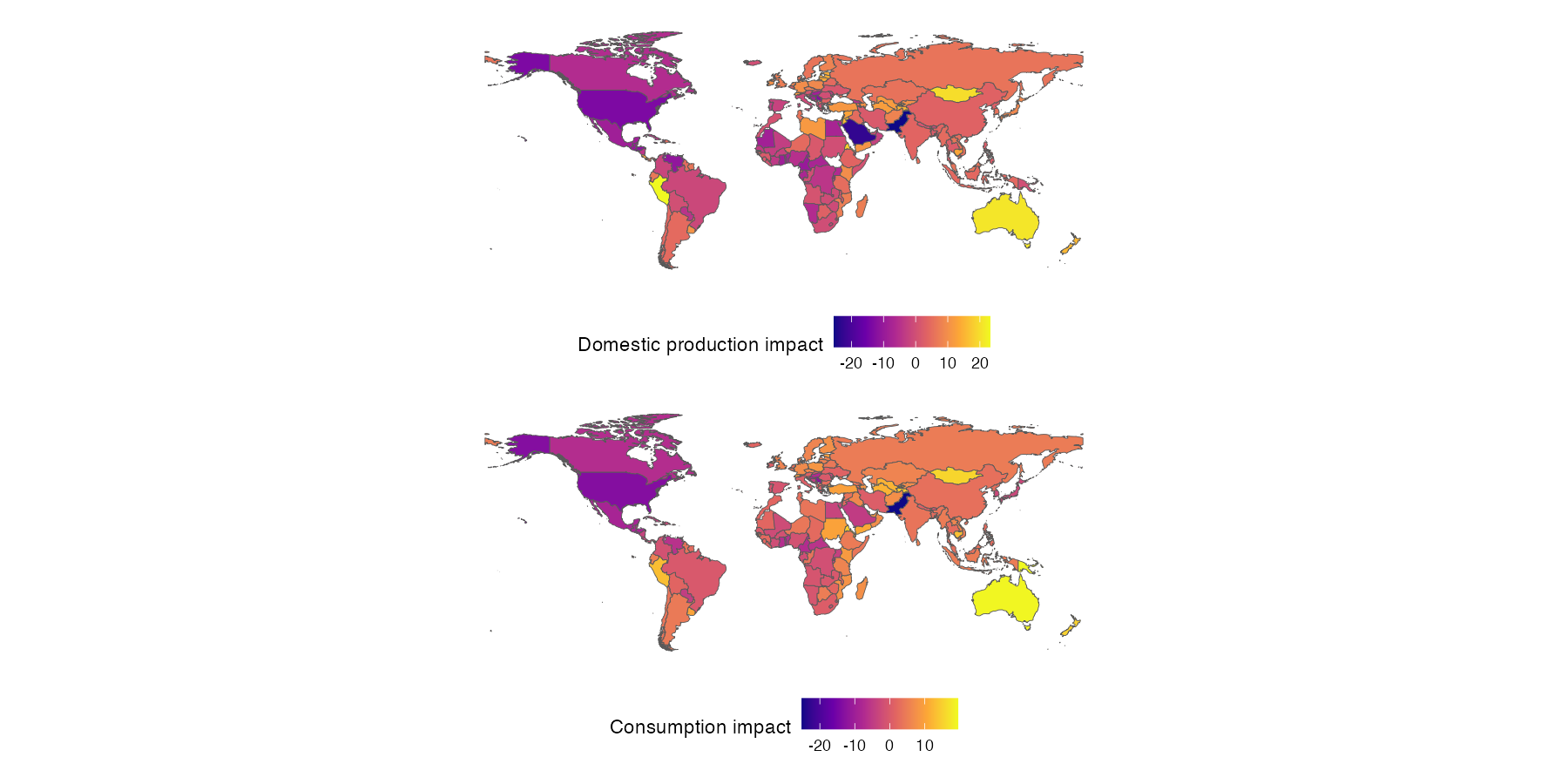

Supplement: S4 Fig — (TIF) [file pone.0314722.s009.tif]

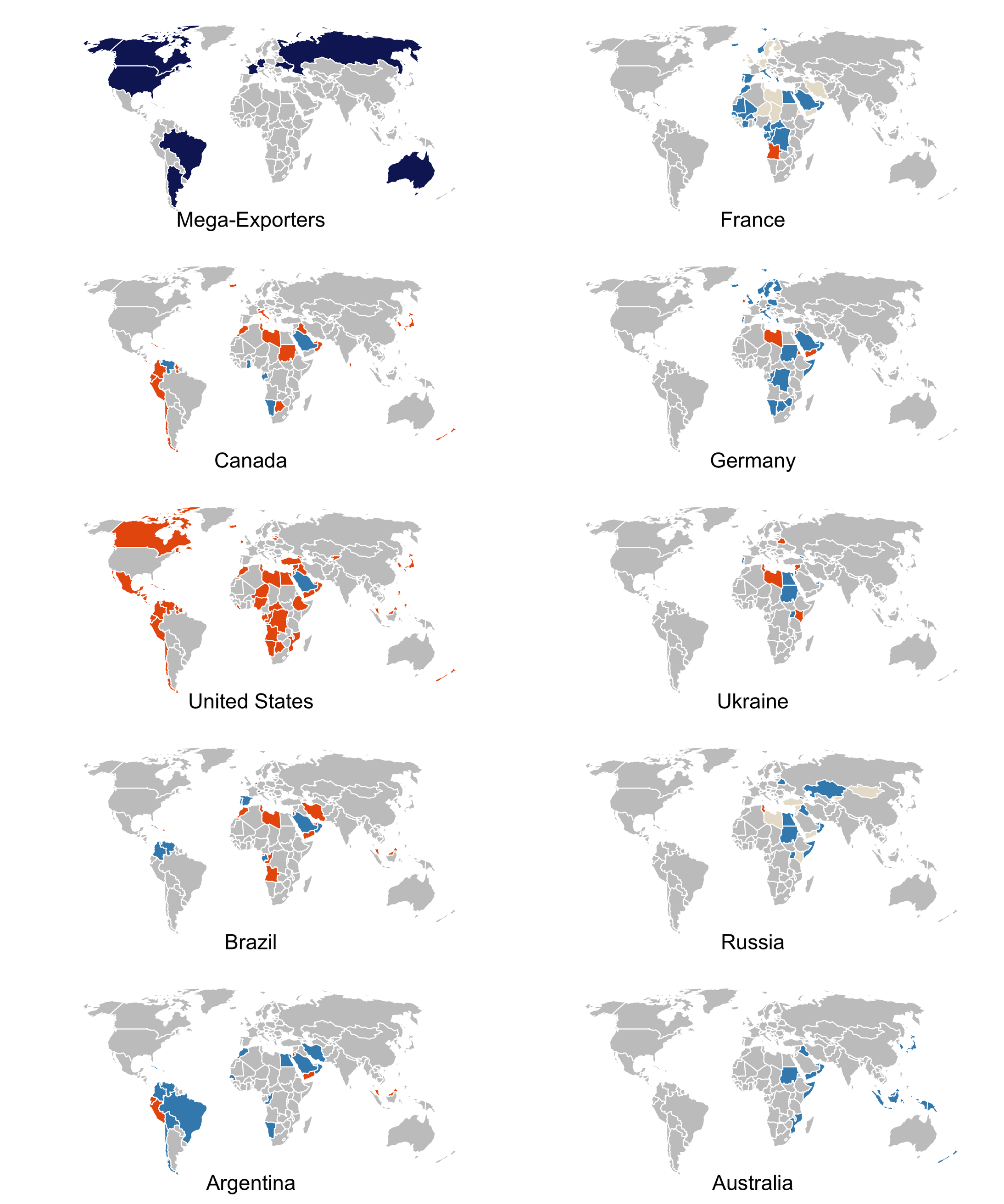

Supplement: S5 Fig — (TIF) [file pone.0314722.s010.tif]

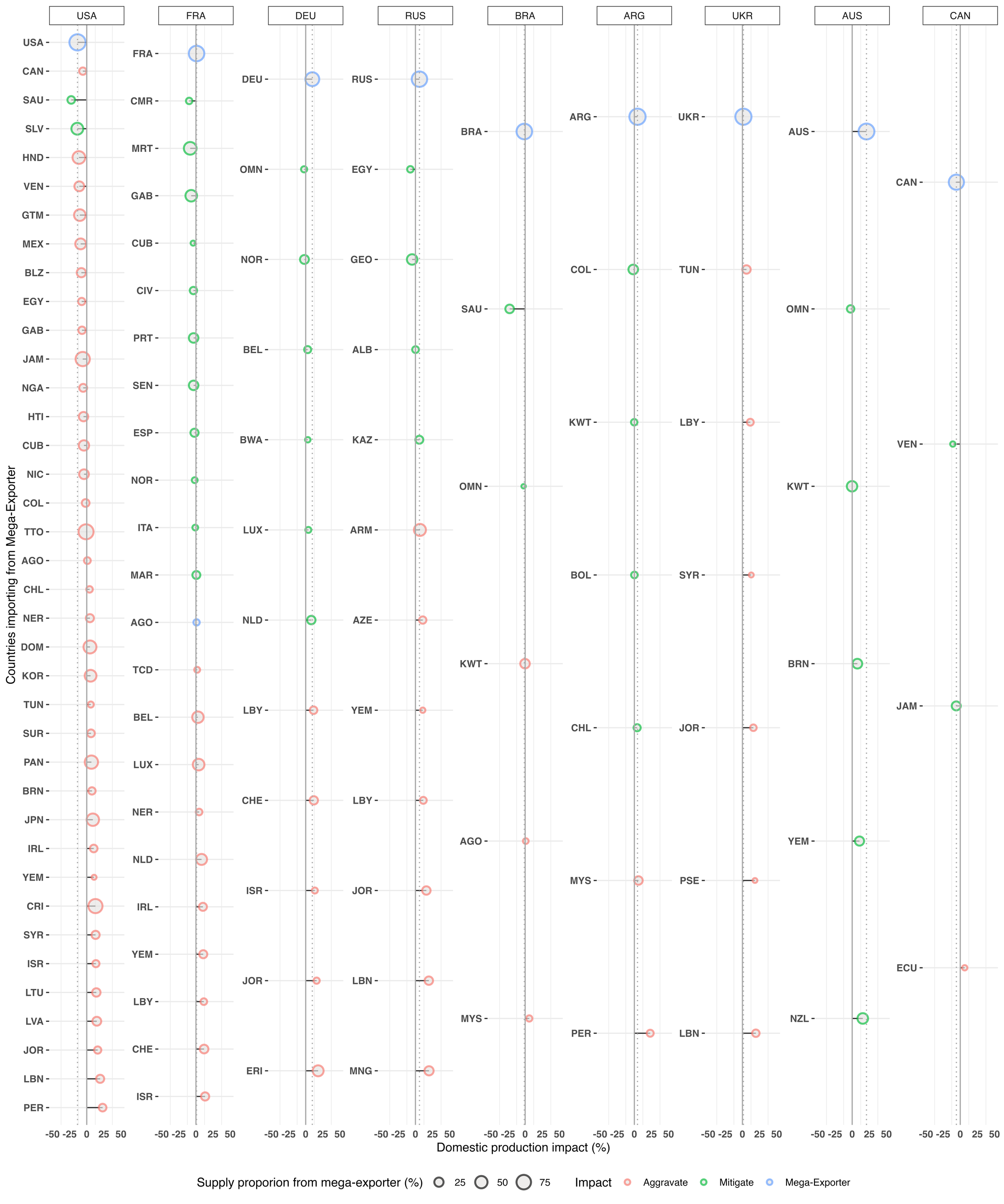

Supplement: S6 Fig — (TIF) [file pone.0314722.s011.tif]

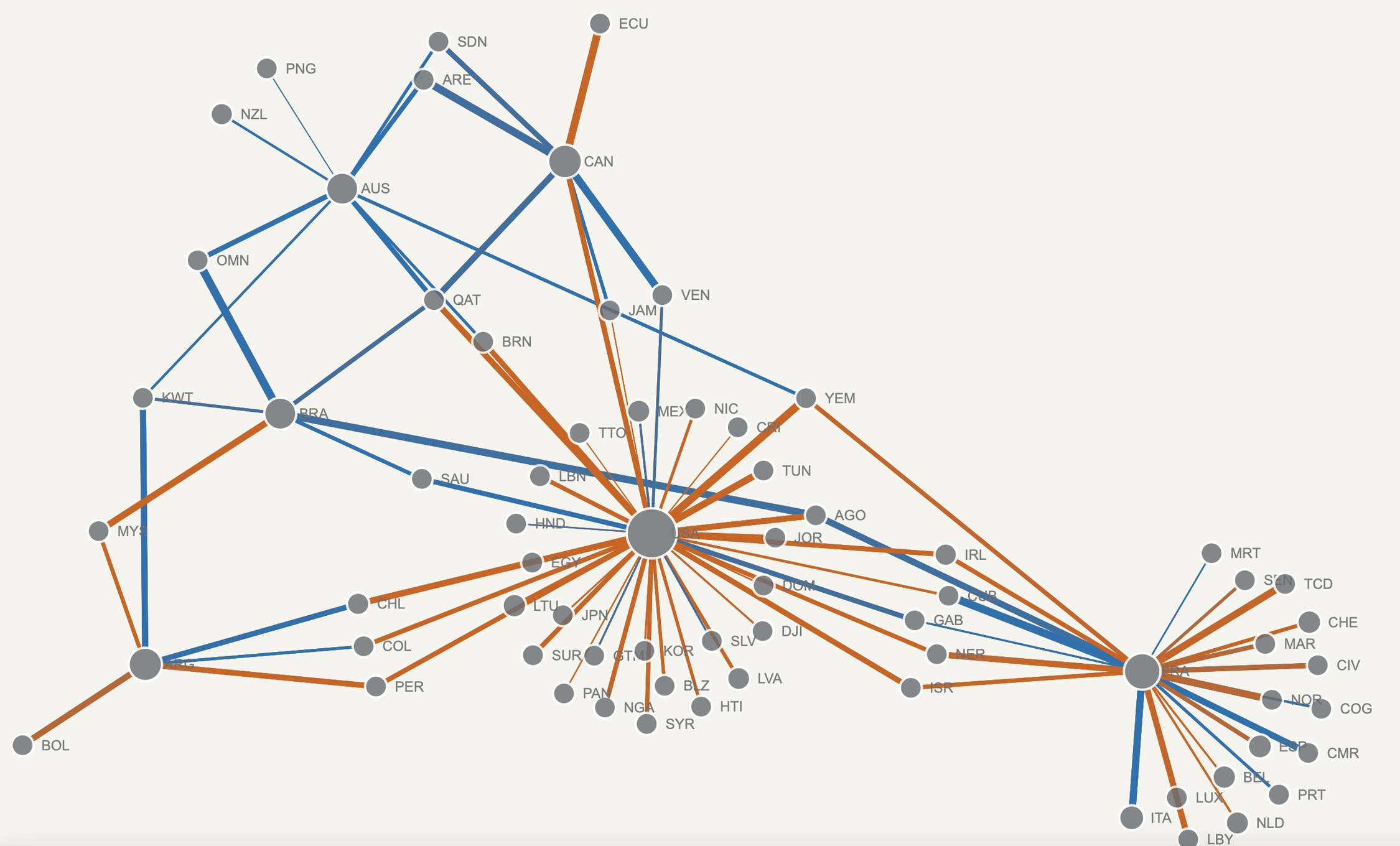

Supplement: S7 Fig — (TIF) [file pone.0314722.s012.tif]

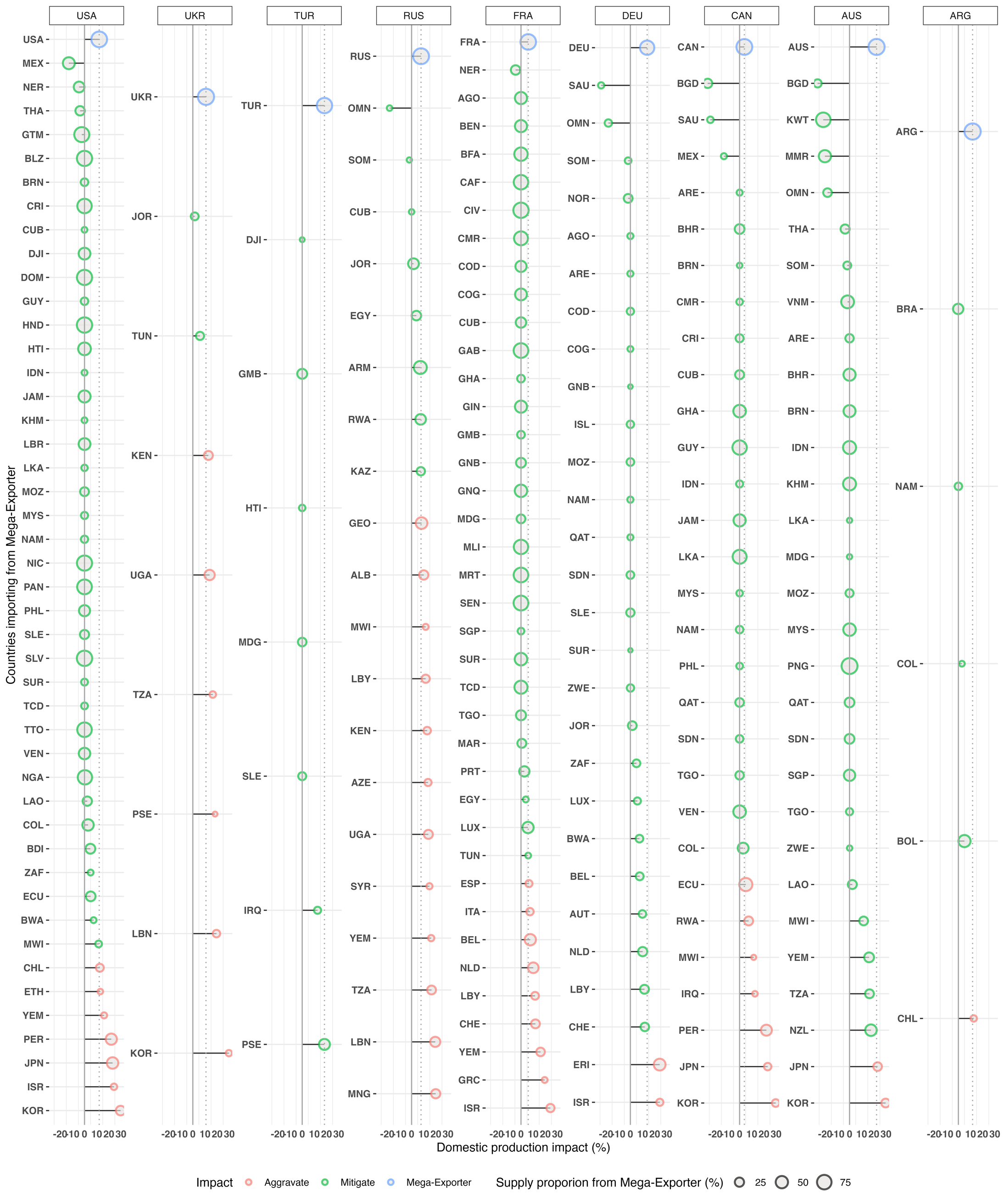

Supplement: S8 Fig — (TIF) [file pone.0314722.s013.tif]

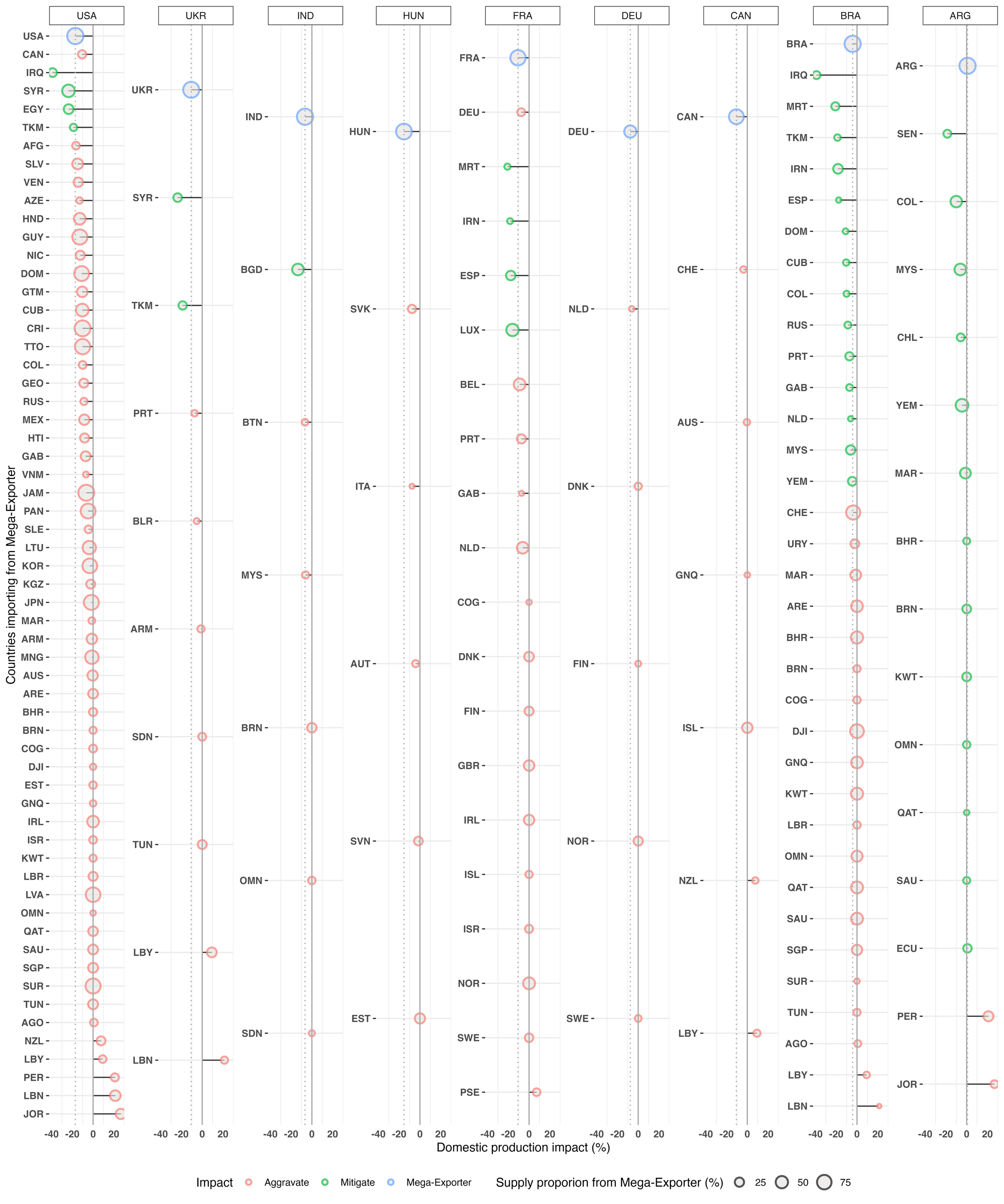

Supplement: S9 Fig — (TIF) [file pone.0314722.s014.tif]

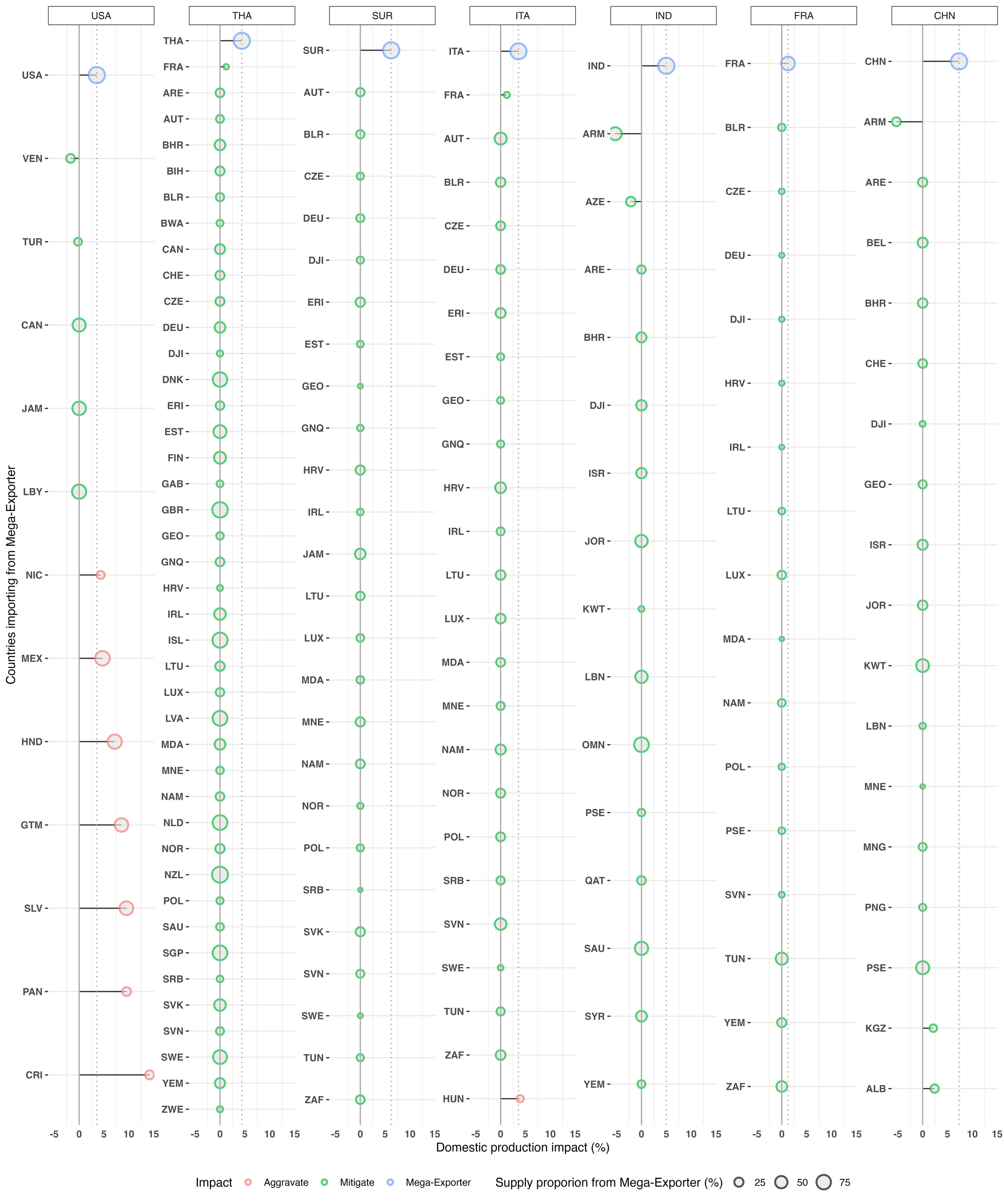

Supplement: S10 Fig — (TIF) [file pone.0314722.s015.tif]

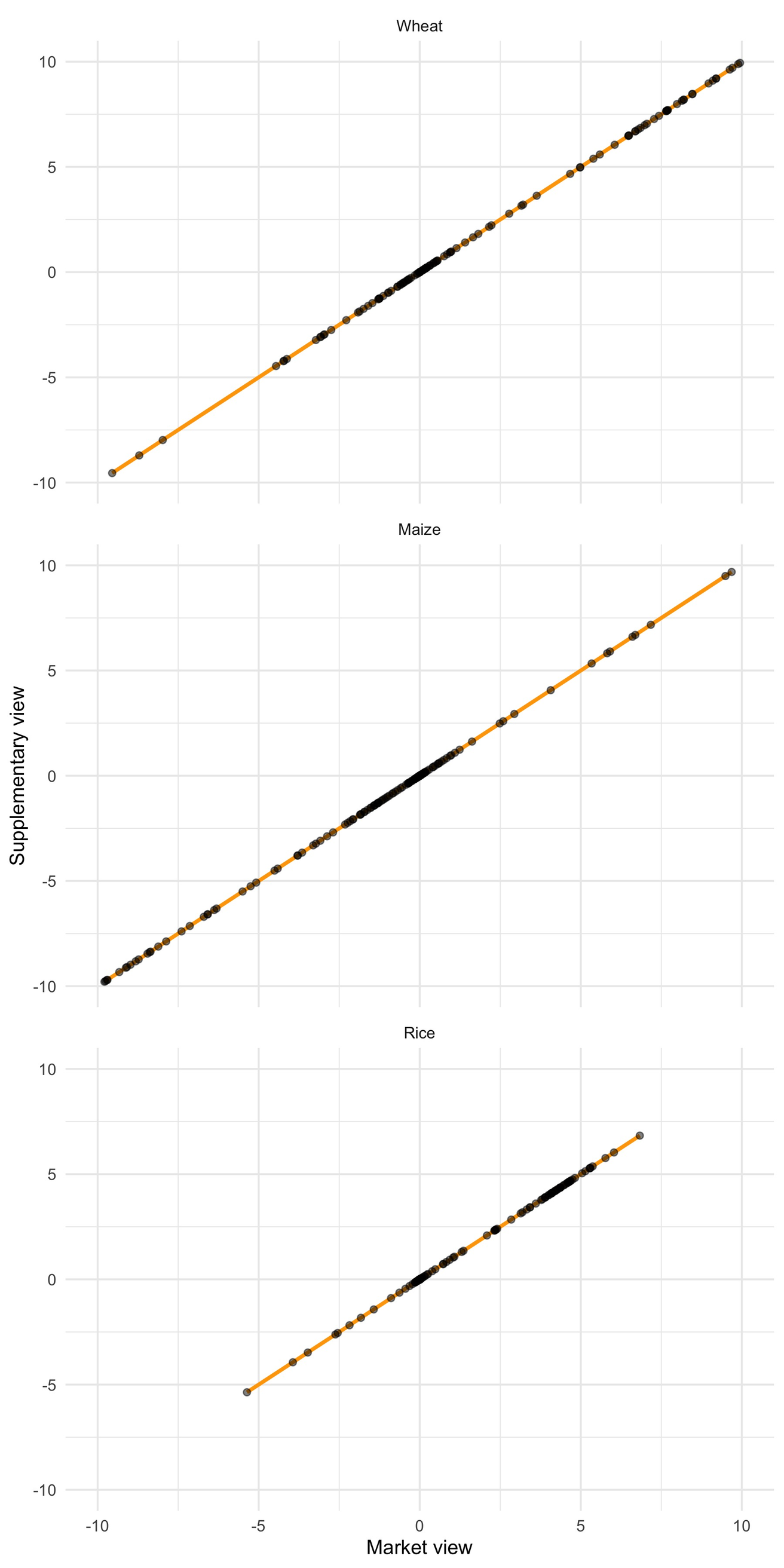

Supplement: S11 Fig — (TIF) [file pone.0314722.s016.tif]
